# The Impact of Prophylactic Negative Wound Pressure Treatment (NWPT) on Surgical Site Occurrences After Gynecologic Cancer Surgery: A Meta-Analysis of Randomized Controlled and Observational Cohort Studies

**DOI:** 10.3390/cancers17101717

**Published:** 2025-05-20

**Authors:** Maximos Frountzas, Ioannis Karavolias, Christina Nikolaou, Orsalia Toutouza, Vasilios Pergialiotis, Konstantinos G. Toutouzas

**Affiliations:** 1 First Propaedeutic Department of Surgery, National and Kapodistrian University of Athens, “Hippocration” General Hospital, 11527 Athens, Greece; ioanniskaravolias@gmail.com (I.K.); tousur@med.uoa.gr (K.G.T.); 2 Department of Plastic and Reconstructive Surgery, Gennimatas General Hospital, 11527 Athens, Greece; kristenni@med.uoa.gr; 3 Medical School, Imperial College, London SW7 2AZ, UK; orsalia.toutouza20@imperial.ac.uk; 4 First Department of Obstetrics and Gynecology, National and Kapodistrian University of Athens, “Alexandra” General Hospital, 11527 Athens, Greece; pergialiotis@yahoo.com

**Keywords:** negative pressure, gynecologic, oncology, surgery, surgical site infections

## Abstract

**Background**: Surgical site infections (SSIs) remain a serious problem following abdominal surgery due to gynecologic malignancies leading to increased hospitalization, high costs, and delays in adjuvant treatments; thus, SSIs affect overall survival. The aim of the present meta-analysis was to investigate the impact of closed incision–negative pressure wound treatment (ci-NPWT) systems on postoperative surgical site occurrences (SSOs) after gynecologic oncology surgery. **Methods**: The present meta-analysis was designed using the PRISMA guidelines. A search in several databases was conducted from inception until March 2025. **Results**: Overall, five studies were included; these studies enrolled 1174 patients in total, where 412 were treated with ci-NPWT systems and 762 were treated with conventional gauze. Patients treated with ci-NPWT systems presented with lower SSI rates (OR 0.40, 95% CI 0.15–1.10, *p* = 0.08), lower fascial dehiscence rates (OR 0.72, 95% CI 0.21–2.42, *p* = 0.59), and lower seroma formation rates (OR 0.70, 95% CI 0.25–1.93, *p* = 0.49), although statistical significance was not reached in all comparisons. On the other hand, patients treated with ci-NPWT systems also presented with higher postoperative hematoma formation rates (OR 1.38, 95% CI 0.32–5.99, *p* = 0.66), although statistical significance was not reached. Preoperative patient characteristics, operative parameters, and cancer characteristics were similar among the two study groups. **Conclusions**: The prophylactic use of ci-NPWT systems showed promising results in reducing postoperative SSOs after gynecologic cancer surgery. Nevertheless, prospectively designed studies are needed in the future to reach robust evidence that would enable the wide implementation of such devices in routine clinical practice.

## Simple Summary

Oncologic outcomes of gynecologic cancer patients undergoing surgery could be influenced by several factors including postoperative complications. Surgical wound infections still remain an important postoperative side effect even after gynecologic oncological operations. Their incidence has been related to patient-related and wound-related risk factors. However, not several preventive measures have been proved efficient yet. Negative pressure wound treatment systems have been implicated to play a potential role in preventing such postoperative complications. Our meta-analysis demonstrated lower wound-related postoperative complications in patients treated with negative pressure wound treatment systems compared to conventional gauze after gynecologic oncologic surgery. Nevertheless, these comparisons did not reach statistical significance. Under these circumstances, prospective randomized trials should be designed to investigate the impact of negative pressure wound treatment on preventing surgical wound infections after gynecologic oncology surgery.

## 1. Introduction

Gynecologic malignancies include a wide spectrum of entities which lead to significant morbidity and mortality. The most common gynecologic malignancy is endometrial cancer, which accounts for approximately 67,880 new cases and an estimated total of 13,250 deaths each year [1,2]. The incidences of cervical and ovarian cancer are reported to be 18.8 and 11.8 per 100,000 people in a population, respectively, with 5-year survival rates of 64.1% and 43%, respectively [3]. The mainstay of treatment for gynecologic malignancies is surgical excision, which often requires complex operations with significant morbidity. Surgical site occurrences (SSOs) are common postoperative complications that occur after gynecologic cancer surgery, including surgical site infection (SSI), fascial dehiscence, hematoma, and seroma, with an overall incidence of 40–60% [4].

The specific incidence of SSIs following surgery treating endometrial cancer, which is the most common among gynecologic cancers, is estimated to be around 30%; SSIs lead to increased length of stay, higher probability of readmission and reoperation, and increased economic burden for the healthcare systems; in terms of costs, each case costs a mean of USD 11,000. Thus, SSIs predispose this issue to a major morbidity factor and hinder the rehabilitation process [5]. Specific types of gynecological cancer, such as cervical cancer, have been theorized to be related to a higher risk for SSI [6]. Among SSIs, superficial ones constitute two thirds of all cases [7]. A prospective audit held in the United Kingdom (UK) enrolled 339 women undergoing laparotomy due to gynecologic cancer; the findings indicated that almost 30% of women diagnosed with an SSI had their adjuvant treatment delayed or cancelled [8]. This could result in a significant impact on overall survival (OS), as demonstrated by a prospective study conducted in the United States of America (USA) which enrolled 8549 patients; participants received adjuvant chemotherapy at a median of 35 days from surgery in the treatment of stage I epithelial ovarian cancer (as assessed by the International Federation of Gynecology and Obstetrics (FIGO)). Patients who experienced a delay had worse OS compared to those who did not (*p* < 0.001; 5-year OS rates of 85.7% and 89.7%, respectively) [9].

The need to counteract such modifiable morbidity factors led to the implementation of closed incision–negative wound pressure therapy (ci-NPWT) systems for the management of closed surgical incisions. Subsequently, the prophylactic use of such alternatives was conceptualized in other surgical disciplines, showing promising results in preventing SSIs and leading to the proposed consensus about their use [10,11,12]. Therefore, the aim of the current meta-analysis is to investigate the impact of ci-NPWT systems on postoperative SSOs after surgery in the treatment of gynecologic cancer; the results for ci-NPWT systems are compared to those for conventional dressing coverage approaches. The findings support the potential of ci-NPWT systems to be the standard treatment option for high-risk patients and abdominal incisions.

## 2. Material and Methods

### 2.1. Protocol and Registration

The present meta-analysis was designed according to the Preferred Reporting Items for Systematic Reviews and Meta-Analyses (PRISMA) recommendations [13]. This study was based on aggregated data that have been previously published in the international literature. Neither patient consent nor institutional review board approval were retrieved as they are not required in this type of study. Our meta-analysis was registered in the Open Science Framework (http://www.osf.io/) as an open-ended registration without a fixed endpoint (unique identifying number: 10.17605/OSF.IO/T2BUJ).

### 2.2. Study Types

Eligibility criteria were predefined by the authors of the reviewed studies and no data restrictions were applied during our literature search. Randomized clinical trials (RCTs) and observational studies (prospective and retrospective) of adult women who underwent abdominal surgery due to several primary and metastatic gynecologic malignancies (such as ovarian, cervical, endometrial, or vulvar cancer) were included. Moreover, another predefined requirement for inclusion was that studies must present a comparison between ciNWPT system treatments and conventional gauze placement; additionally, we required that the studies reported on postoperative surgical site occurrences (SSOs), such as dehiscence, surgical site infection (SSI), hematoma, and seroma. All articles written in the Latin alphabet were included in the present study, as well as articles written in other languages if they could be translated using the Google Translate service. Case reports, experimental animal studies, and reviews were excluded from the present study.

### 2.3. Information Sources and Search

**Figure 1:**
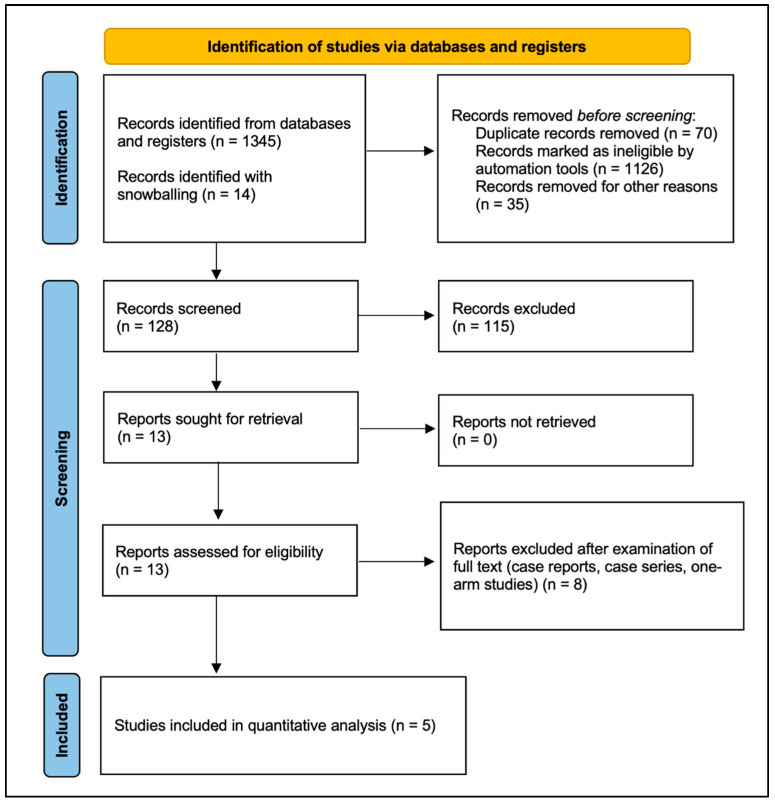
The PRISMA 2020 flowchart for the included studies.

Medline (1966–2025), Scopus (2004–2025), EMBASE (1980–2025), Clinicaltrials.gov (2008–2025), Google Scholar (2004–2025), and Cochrane Central Register of Controlled Trials CENTRAL (1999–2025) were explored by two of the authors to identify qualifying research published before 10 March 2025. These databases are the most famous in medical and scientific research; thus, in choosing these databases, we maximize the likelihood that we will include all the available evidence required to address the primary research question of the present meta-analysis. Please note that the special characteristics of several of the databases (e.g., the uncontrolled content of Google Scholar) were considered during the data extraction process. This process was conducted by two experienced authors. In addition, the chance of including all available articles that met the inclusion criteria was maximized by consulting the references in the articles that were retrieved in full text. Any disagreements between reviewers were resolved by a third reviewer. The main search strategy was as follows: (“prophylactic” OR “closed incision”) AND “negative pressure wound therapy” AND (“gynecologic” OR “ovarian” OR “endometrial” OR “cervical” OR “vulvar”) AND “cancer”. We applied no filters regarding study type, language, or publication time. The article selection process is demonstrated in the PRISMA flow diagram (Figure 1).

Study selection was performed in three consecutive stages: First, duplicate publications were removed, and the titles and abstracts of all the electronic articles that were retrieved in the search were evaluated in terms of eligibility. Second, the full texts of all articles that met the inclusion criteria were downloaded and all prospective and retrospective observational studies, along with randomized controlled trials, were selected. Predefined exclusion criteria were applied at each stage of the data extraction process. Study search and data tabulation was conducted by two experienced authors on similar predefined forms, following strict predefined rules. The two data extractors were blinded to the other’s extraction outcomes to minimize bias. In cases of discrepancies, the first step was rechecking the original study data, followed by the participation of a third more experienced reviewer if necessary. Finally, consensus among all authors resolved any possible conflicts after all available data were retrieved.

### 2.4. Study Data and Predefined Outcomes

Table 1 contains the number of patients included in each study, the type of study, the country where each study was conducted, the inclusion and exclusion criteria for each study, and the follow-up intervals for each study. Table 2 depicts the enrolled patients’ data: age, body mass index (BMI), smoking status, diabetes mellitus, cardiovascular disease, history of steroid use, previous surgeries, and performance status according to American Society of Anesthesiologist (ASA) classification. Table 3 includes operative parameters, such as blood loss, operative duration, bowel resection, blood transfusion, staple skin closure, and wound classification according to Center of Disease Control (CDC) classifications. The malignancy characteristics of the enrolled patients, such as the site of cancer, their disease stage according to the FIGO classification, and neoadjuvant treatment administration, are depicted in Table 4. Finally, Table 5 describes postoperative outcomes, such as surgical site infection (SSI), fascial dehiscence, seroma, hematoma, and length of stay.

The outcomes of the studies which were investigated in the present meta-analysis were pre-determined. Surgical site infections (SSIs) were predefined as the primary outcomes. On the other hand, secondary outcomes included the fascial dehiscence rate, the seroma and hematoma rate, as well as the postoperative length of stay interval.

### 2.5. Risk of Bias Assessment

The methodological quality of included RCTs was assessed by two independent reviewers using the risk of bias 2 (RoB2) tool [19]. The quality of non-randomized trials was assessed by two independent reviewers with the Risk of Bias in Non-Randomized Trials (RoBINS-I) tool [20].

### 2.6. Statistical Analysis

Statistical analysis was performed using the RevMan 5.3 software (Copenhagen: The Nordic Cochrane Centre, The Cochrane Collaboration, 2011). The confidence interval (CI) level was assumed at 95%. A random-effects model (DerSimonian-Laird) using arcsine square root (Freeman–Tukey) transformation was utilized to derive pooled odds ratios (OR) and the 95% CIs [21]. A random-effects model was utilized due to substantial heterogeneity among the included studies and provided a more conservative statistical estimate, incorporating both within-study and between-study variability. Moreover, the DerSimonian–Laird estimator was used to estimate the between-study variance and calculate the overall effect size, while accounting for both within-study and between-study variability. The arcsine square root transformation was used to stabilize variance and render the data more suitable for analysis, especially for our meta-analysis which included proportion data [22]. Study heterogeneity was estimated according to the inconsistency index (*I*^2^) [23]. A significance level of *p* < 0.05 and *I*^2^ value of ≥50% indicated high heterogeneity.

## 3. Results

### 3.1. Included Studies

**Figure 2:**
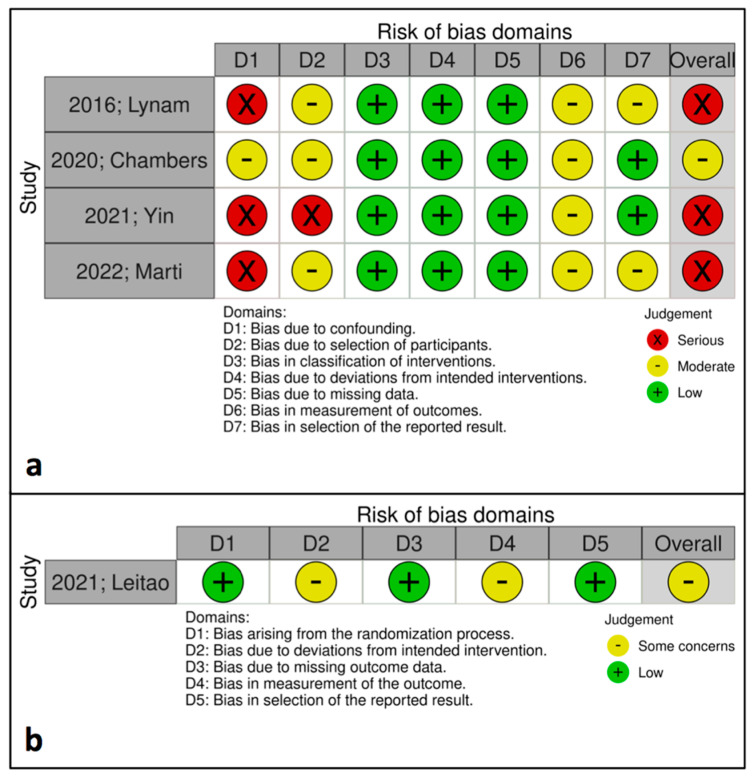
Risk of Bias in Non-Randomized Trials (RoBINS—I) assessment of included observational studies [14,15,17,18] (**a**); Risk of bias 2 (RoB2) assessment of randomized trials [16] (**b**).

In total, five studies were included in the present meta-analysis, which enrolled a total of 1174 patients [14,15,16,17,18]. A ci-NPWT system was utilized in the treatment of 412 patients undergoing abdominal surgery due to gynecologic cancer; a conventional gauze was used to cover the surgical incision during the treatment of 762 patients (Table 1). Three of the included studies [14,15,18] were retrospective cohort trials; one study [17] was a prospective observational trial; one study [16] was a randomized controlled trial. Three studies [14,15,16] were conducted in the United States of America (USA), while one study [17] was performed in the United Kingdom (UK) and one study was performed in Spain [18]. Moreover, the quality assessment of the observational studies according to the RoBINS-I tool indicated that three out of four studies [14,17,18] had serious risk of bias; one study [15] was found to have a moderate risk of bias (Figure 2). Furthermore, the RoB2 tool which was utilized for the evaluation of the RCT [16] demonstrated that the risk of bias was moderate for this study (Figure 2). The postoperative follow-up interval was 30 days for all studies [15,16,17,18]; the exception to this is Lynam et al.’s study, in which the included patients were followed-up with 90 days after surgery [14]. All studies utilized ci-NPWT systems with a constant pressure of −125 mmHg.

### 3.2. Quantitative Analysis

**Figure 3:**
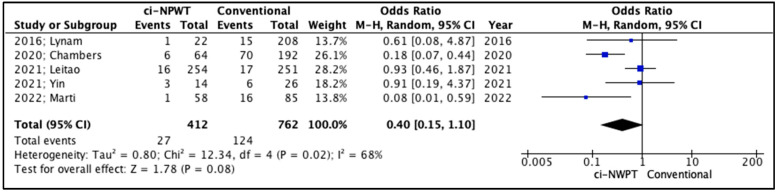
Forest plot analysis of surgical site infections (SSIs): vertical line, no difference point between two groups; squares, odds ratios; diamonds, pooled odds ratio for all studies; horizontal lines, 95% CI [14,15,16,17,18].

The occurrence of surgical site infections (SSIs) after surgery in the treatment of gynecologic cancer was predefined as the primary outcome for the present study (Table 5). SSIs were lower in patients treated with ci-NPWT systems compared to patients treated conventionally with a gauze covering the surgical incision, but this comparison did not reach statistically significant levels (OR 0.40, 95% CI 0.15–1.10, *p* = 0.08), whereas the level of inter-study heterogeneity was high (*I*^2^ = 68%, Figure 3).

**Figure 4:**
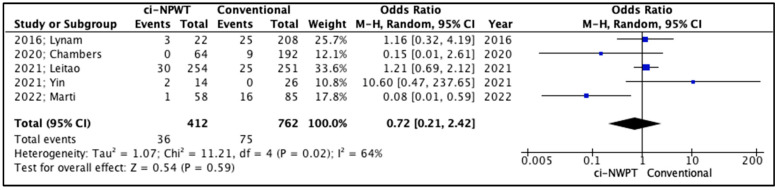
Forest plot analysis of fascial dehiscence: vertical line, no difference point between two groups; squares, odds ratios; diamonds, pooled odds ratio for all studies; horizontal lines, 95% CI [14,15,16,17,18].

**Figure 5:**
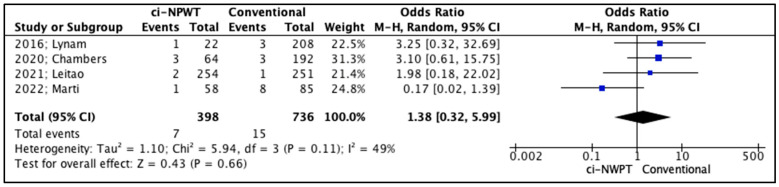
Forest plot analysis of hematoma: vertical line, no difference point between two groups; squares, odds ratios; diamonds, pooled odds ratio for all studies; horizontal lines, 95% CI [14,15,16,18].

**Figure 6:**
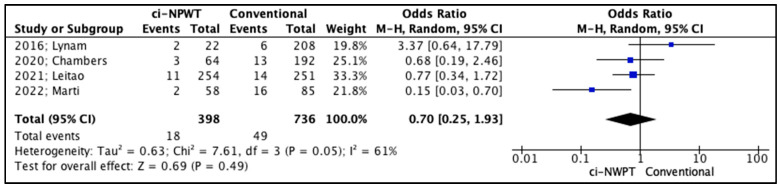
Forest plot analysis of seroma: vertical line, no difference point between two groups; squares, odds ratios; diamonds, pooled odds ratio for all studies; horizontal lines, 95% CI [14,15,16,18].

The secondary outcomes in the studies included fascial dehiscence, hematoma formation, and seroma formation (Table 5). Firstly, fascial dehiscence rates were lower in patients treated with ci-NPWT systems compared to patients treated with conventional gauzes, but the outcomes of this comparison did not reach statistical significance (OR 0.72, 95% CI 0.21–2.42, *p* = 0.59); meanwhile, the level of inter-study heterogeneity was high (I^2^ = 64%, Figure 4). On the other hand, the risk for hematoma formation in patients treated with ci-NPWT systems was higher compared to that for patients treated with conventional gauzes (OR 1.38, 95% CI 0.32–5.99, *p* = 0.66); however, statistical significance was not reached, and the level of inter-study heterogeneity was low (I^2^ = 49%, Figure 5). Furthermore, seroma formation was lower in patients treated with ci-NPWT systems compared to those treated with conventional gauzes (OR 0.70, 95% CI 0.25–1.93, *p* = 0.49); however, statistical significance was not reached, and the level of inter-study heterogeneity was high (I^2^ = 61%, Figure 6).

### 3.3. Qualitative Analysis

The included studies did not provide sufficient data for a quantitative analysis to be conducted regarding the postoperative length of stay (Table 5). Lynam et al. reported a similar mean postoperative length of stay between patients treated with ci-NPWT systems and those treated with conventional gauzes (6.22 days vs. 5.25 days, *p* = 0.20) [14]. On the other hand, Marti et al. demonstrated a significantly lower postoperative length of stay in patients treated with ci-NPWT systems compared to patients treated with conventional gauzes (6.16 days vs. 8.86 days, *p* = 0.036) [18].

Patient characteristics (age; BMI; smoking status; diabetes mellitus; cardiovascular disease; steroid use; prior surgery history; performance status according to ASA score) that could be considered to be potential risk factors for adverse wound-related outcomes following abdominal surgery due to gynecologic malignancy were similar among the two study groups (Table 2). Lynam et al. reported significantly higher blood loss for patients treated with ci-NPWT systems compared to patients treated with conventional gauzes (656 mL vs. 394 mL, *p* = 0.02); additionally, they reported higher stapler skin closure rates for patients treated with ci-NPWT systems compared to patients treated with conventional gauzes (86% vs. 51%, *p* < 0.001) [14]. On the other hand, no significant differences were demonstrated in the other included studies concerning intraoperative blood loss or stapler skin closure. The rest of reported operative parameters, such as operative duration, bowel resection, blood transfusion, and CDC wound classification, were similar between the two study groups among the included studies, providing data on related outcomes (Table 3).

Malignancy characteristics, such as cancer site, disease state, and neoadjuvant therapy administration, were similar among patients treated with ci-NPWT systems and patients managed with conventional gauzes (Table 4). However, 564 of the included patients (48%) suffered from ovarian cancer, 171 patients (15%) were diagnosed with endometrial cancer, and 26 patients (2%) suffered from cervical cancer. Moreover, 134 patients (11%) had FIGO stage I disease, 35 patients (3%) had FIGO stage II disease, 193 patients (16%) had FIGO stage III disease, and 89 patients (8%) were diagnosed with FIGO stage IV disease. Finally, 239 patients (20%) had received neoadjuvant chemotherapy, and 15 patients (1%) had received preoperative radiation therapy.

## 4. Discussion

Gynecologic cancer is a common malignancy related to the important aspects of survival burden, especially ovarian and cervical cancer. Surgical management remains the cornerstone of treatment; however, complex operations are usually required, which have been associated with an important aspect—postoperative morbidity. This could affect adjuvant treatment administration and overall survival. The present meta-analysis investigated the potential role of ci-NPWT systems in preventing SSOs after gynecologic cancer abdominal surgery, which might lead to prolonged hospitalization and delays in adjuvant treatment initiation. Demographic characteristics, operative details, and cancer-related parameters demonstrated no significant differences among the included studies; one exception to this is that, in one study, higher blood loss and a higher stapler skin closure rate were noted for the group treated with ci-NPWT compared to the group treated with conventional gauze [14]. Moreover, SSIs were lower in patients treated with ci-NPWT systems after gynecologic cancer surgery compared to patients treated with conventional gauze; this difference almost reached statistical significance. This could be related to the low methodological quality and serious risk of bias that were present in the majority of the studies included in this review. Furthermore, the risk for fascial dehiscence and seroma formation was lower in patients treated with ci-NPWT compared to those treated with conventional gauzes, even though statistical significance was not reached. Finally, the risk for hematoma formation was higher in patients treated with ci-NPWT systems compared to those treated with conventional gauzes; however, this difference did not reach statistical significance either.

### 4.1. Implications of Negative Pressure Wound Therapy

The rationale behind the utilization of ci-NPWT systems in the prevention of SSOs after abdominal operations in the treatment of gynecologic malignancies is based on several high-scale meta-analyses highlighting the potential benefits of such devices after surgery for different indications. Firstly, Groenen et al. reported a significant reduction in SSIs regarding the use of incisional NPWT after abdominal surgery compared to conventional incisional management (7.9% vs. 11.6%, RR = 0.67, 95% CI 0.59–0.76, *p* < 0.001) in a meta-analysis and trial sequential analysis, including 13,744 patients from 57 RCTs [24]. Such findings were also confirmed by another meta-analysis of 19 RCTs and observational studies, which indicated an overall SSI reduction (OR = 0.36, 95% CI 0.27–0.49, *p* < 0.001) in patients utilizing single-use NPWT compared to conventional dressings in closed surgical incisions after abdominal surgery. This reduction was noticed both in superficial SSIs (OR = 0.30, 95% CI 0.17–0.53, *p* < 0.001) and deep SSIs (OR = 0.67, 95% CI 0.46–0.96, *p* < 0.001) [25]. The most comprehensive meta-analysis in the field accounted for 23,546 patients enrolled in 84 studies; the authors investigated the impact of ci-NPWT compared to standard of care after cardiac, abdominal, obstetric, orthopedic, plastic, and vascular surgery. It demonstrated a significant reduction in overall SSO (RR = 0.543, *p* < 0.001) and SSI rates (RR = 0.530, *p* < 0.001), reduced superficial SSIs (RR = 0.505, *p* < 0.001), deep SSIs (RR = 0.469, *p* = 0.002), seroma formation (RR = 0.677, *p* = 0.004), fascial dehiscence rates (RR = 0.644, *p* = 0.022), and skin necrosis (RR = 0.466, *p* = 0.001) rates, as well as lower readmission rates (RR = 0.773, *p* = 0.039), reoperation rates (RR = 0.64, *p* < 0.001), and postoperative pain (*p* < 0.001) [26].

Apart from the prevention of SSOs, NPWT seems to have promising results in the management of adverse wound events following gynecologic surgery. A retrospective study from the USA highlighted the efficacy of NPWT in managing complex failed wounds after gynecologic oncology surgery, such as total abdominal hysterectomy with bilateral salpingo-oopherectomy, vulvectomy with or without inguinal lymph node dissection, skin or myocutaneous grafting, parastomal herniorrhaphy, and retroperitoneal lymph node dissection. The use of NPWT was associated with an overall reduction of 96% (range: 0–100%) in the median size of wound defects, from 330 to 14 cm^3^, with a complete wound healing rate of 96% [27]. Furthermore, the potential benefit of NPWT in managing failed wounds after gynecologic surgeries has been demonstrated in several case series involving soft tissue necrosis after Pfannenstiel incision, post-hysterectomy abdominal wound dehiscence, and necrotizing fasciitis after cesarean delivery [28,29,30].

### 4.2. Strengths and Weaknesses

To the best of our knowledge, this is the first meta-analysis which presents a quantitative comparison regarding the impact of ci-NPWT systems on postoperative-wound-related outcomes after surgery due to gynecologic malignancies compared to the standard of practice with conventional dressings, including studies with two-arm comparisons. Furthermore, wide inclusion criteria were applied during the search procedure; hence, we maximized the likelihood of including all the available studies that are related to our topic. The search and selection of the included studies were performed by two independent researchers, limiting selection bias. Another advantage is the predefined methodological protocol which was followed during the interpretation of the present meta-analysis; this is registered in an international database (Open Science Framework), giving every reviewer the opportunity to assess its methodological quality and statistical adequacy. Last but not least, the methodological quality of the included studies was assessed using two separate tools for observational cohort studies (RoBINS-I) and randomized controlled trials (RoB2), indicating the specific risk of bias for each included study.

On the other hand, the present meta-analysis has several limitations. First of all, the total number of patients enrolled is small; this is because the included studies provided adequate comparisons between ci-NPWT and conventional dressings, as well as postoperative-wound-related outcomes for only small cohorts of patients. In addition, the included studies present a high level of heterogeneity which was counterbalanced by the utilization of the random-effects model (DerSimonian-Laird) to derive the pooled values of outcomes (ORs and 95% CIs). This heterogeneity could be counterbalanced by subgroup analyses regarding specific cancer types (ovarian, endometrial, cervical) or patient-related high-risk factors. Nevertheless, the included studies did not provide postoperative data regarding the use of ci-NPWT systems stratified according to such covariates. Finally, the majority of the included studies were observational retrospective cohort trials, which lower the overall quality of the quantitative analysis; this is indicated by the methodological quality assessment according to RoBINS-I and RoB2 tools, which showed a serious risk of bias for three out of the five included studies. The prospective design of future studies might overcome this methodological drawback and facilitate statistically powered comparisons that would benefit clinical practice.

### 4.3. Clinical Practice and Future Research

SSIs are the most common hospital-acquired infections worldwide, leading to increased length of stay, higher readmission rates, increased pathogen resistance due to wider antibiotic administration, destruction of cometic impact and patient satisfaction, increased hospital costs, and worsening of cancer survival due to delays in starting adjuvant after surgery [31]. Under these circumstances, the World Health Organization (WHO) has published a prevention bundle for SSIs, which includes prophylactic NPWT as an intraoperative measure for reducing SSIs [32]. According to the only available consensus so far, ci-NPWT is recommended in patients undergoing abdominal surgery with high-risk features for SSIs (e.g., diabetes, obesity, hypoalbuminemia, chronic renal insufficiency, chronic obstructive pulmonary disease, current or recent cessation of tobacco use, corticosteroid use, or recent chemotherapy). Moreover, the use of ci-NPWT is recommended in incisions with high-risk features for SSIs, such as repeated incisions or revision surgeries, extensive undermining, traumatized soft tissue, edema, preoperative radiation therapy, post-bariatric abdominoplasty, soilage risk, compromised perfusion, and high tension [33].

The use of ci-NPWT is accompanied by a higher primary cost than the conventional dressings. Therefore, their use should be tailored to high-risk patients and high-risk abdominal incisions, as mentioned above. A detailed, informative approach is indicated to overcome any potential resistance among patients regarding speculated intolerance of NPWT devices, as ci-NPWT systems are smaller than the conventional NPWT devices; they are also lighter and portable, with battery autonomy for several hours or days. In addition, the present meta-analysis indicated a promising trend towards the efficacy of such devices in preventing SSI, fascial dehiscence, and seroma formation after abdominal surgery due to gynecologic cancer. However, statistically significant comparisons were not reached. This limitation could be overcome in the future by conducting large-scale studies with prospective designs. Observational or randomized clinical trials would increase the methodological quality of future comparisons, providing proof for the impact of ci-NPWT systems in preventing SSOs after gynecologic oncology surgery and facilitating their implementation in clinical practice with robust evidence.

## 5. Conclusions

SSIs after abdominal surgery in the treatment of gynecologic malignancy remain a serious problem; they increase hospital costs and could burden overall survival due to delays in starting adjuvant therapy. Among the multiple strategies for preventing SSIs after gynecologic cancer surgery, ci-NPWT seems to have a beneficial impact on reducing SSIs, fascial dehiscence, and seroma formation. However, considering the high cost of utilization, a tailored approach should be implemented based on patient-related or incision-related risk factors. In addition, prospectively designed observational or randomized trials are required to produce robust evidence regarding the preventive use of such devices in the field of gynecologic oncology surgery.

## Figures and Tables

**Table 1:** The characteristics of the included studies. ci-NPWT, closed incision–negative pressure wound therapy; USA, United States of America; UK, United Kingdom; BMI, body mass index; N/A, not available.

Study Characteristics (ciNWPT vs. Conventional Gauze)						
*Year; Author*	*Type of Study*	*No. of Patients (ciNWPT* vs. *Conventional Gauze)*	*Study Origin*	*Inclusion Criteria*	*Exclusion Criteria*	*Postoperative Follow-up*
2016; Lynam [14]	Retrospective case–control study	230 (22 vs. 208)	USA	High-risk for wound complications	Lost to follow-up	90 days
2020; Chambers [15]	Retrospective multicenter case–control study	256 (64 vs. 192)	USA	Surgeons’ estimation of high risk	Laparotomy for tissue extraction	30 days
2021; Leitao [16]	Randomized single blinded clinical trial	505 (254 vs. 251)	USA	Women aged ≥ 18 years with gynecologic malignancy; BMI ≥ 40 kg/m^2^; Benign indication.	Open abdomen	30 days
2021; Yin [17]	Observational cohort study	40 (14 vs. 26)	UK	Gynecological oncology; Midline laparotomy incision; Vacuum dressing after primary closure.	Transverse incision laparotomies; Laparoscopic surgeries with incisions below the umbilicus; Vacuum dressings for late management of postoperative wound breakdown.	30 days
2022; Marti [18]	Retrospective cohort study	143 (58 vs. 85)	Spain	Age older than 18 years; Confirmed gynecologic malignancy; Cytoreductive surgery with midline laparotomy; Written informed consent.	Benign pathology; No consent; Minimally invasive surgery.	30 days

**Table 2:** Demographic characteristics of enrolled patients. ci-NPWT, closed incision–negative pressure wound therapy; CAD, coronary artery disease; MI, myocardial infarction; VTE, venous thromboembolism; ASA, American Society of Anesthesiologists; N/A, not available.

Patient Characteristics (ciNWPT vs. Conventional Gauze)									
*Year; Author*	*Age (Years)*	*BMI (kg/m^2^)*	*Smoking*	*Diabetes*	*Cardiovascular Disease*	*Steroids*	*Previous Surgery*	*Performance Status (ASA Score)*	
								*ciNWPT*	*Conventional Gauze*
2016; Lynam [14]	54.9 vs. 53.2	41.29 vs. 30.67	5 (22.73%) vs. 30 (14.42%)	7 (31.82%) vs. 35 (16.83%)	N/A	0 vs. 6 (2.88%)	12 (54.54%) vs. 70 (33.65%)	N/A	N/A
2020; Chambers [15]	59.0 ± 11.8 vs. 60.9 ± 11.8	<30: 14 (21.9%) vs. 51 (26.6%) 31–40: 27 (42.2%) vs. 85 (44.3%) 41–50: 19 (71.9%) vs. 52 (27.1%) >51: 4 (6.3%) vs. 4 (2.1%)	Current: 4 (6.2%) vs. 16 (0.8%) Historical: 19 (29.2%) vs. 40 (20.9%) None: 42 (64.6%) vs. 135 (70.7%)	25 (39%) vs. 75 (39.1%)	CAD: 7 (10.9%) vs. 22 (11.5%) Prior MI: 1 (1.6%) vs. 2 (1.0%) Prior Stroke: 0 vs. 8 (4.2%) Prior VTE: 11 (17.2%) vs. 25 (13%)	2 (3.1%) vs. 3 (1.6%)	N/A	(1) 7 (10.9%) (2) 20 (31.3%) (3) 36 (56.3%) (4) 1 (1.6%)	(1) 1 (0.5%) (2) 40 (20.8%) (3) 141 (73.4%) (4) 10 (5.2%)
2021; Leitao [16]	60 (20–85) vs. 61 (23–87)	26 (range, 18–60) vs. 26 (range, 17–56)	Never: 143 (57%)/152 (61%) Current: 10 (4%)/11 (4%) Former: 97 (39%)/87 (35%)	36 (14%) vs. 0	Hypertension: 85 (34%) vs. 86 (35%) Vascular disease: 7 (2.8%) vs. 12 (5%)		175 (70%) vs. 168 (68%)	N/A	N/A
2021; Yin [17]	59.6 vs. 57.6	≥30: 5 (36%) vs. 4 (15%)	Active smoking 5 (36%)/4 (15%)	3 (21%) vs. 1 (4%)	N/A	N/A		ASA grade ≥ 3:10 (71%)	ASA grade ≥ 3:6 (23%)
2022; Marti [18]	63.28 vs. 61.51	28.59 vs. 27.59	N/A	12 (20.7%) vs. 19 (22.4%)	N/A	N/A	31 (53.4%) vs. 50 (58.8%)	N/A	N/A

**Table 3:** Operative parameters of enrolled patients. ci-NPWT, closed incision–negative pressure wound therapy; CDC, Center of Disease Control; N/A, not available.

Operative Parameters (ciNWPT vs. Conventional Gauze)						
*Year; Author*	*Blood Loss (mL)*	*Operative Duration (min)*	*Bowel Resection*	*Blood Transfusion*	*Staple Closure*	*Wound Classification (CDC)*
2016; Lynam [14]	656 vs. 394	138 vs. 137	0 vs. 34 (16.34%)	6 (27.27%) vs. 61 (29.19%)	19 (86.36%) vs. 106 (51%)	N/A
2020; Chambers [15]	N/A	233.0 (range, 136.5–311.5) vs. 211.0 (range, 150–313.0)	N/A	N/A	53 (82.8%) vs. 147 (76.6%)	Mean 2.0 vs. 2.0
2021; Leitao [16]	400 (range, 5–3200) vs. 300 (range, 5–3300)	291 (range, 56–701) vs. 256 (range, 60–786)	92 (37%) vs. 92 (37%)	46 (18%) vs. 31 (12%)	254 (100%) vs. 251 (100%)	Clean 16 (6%) vs. 11 (4%) Clean-contaminated 229 (91%) vs. 236 (94%) Contaminated/Dirty 6 (2.4%) vs. 3 (1.2%)
2021; Yin [17]	N/A	N/A	N/A	N/A	14 (100%) vs. N/A	N/A
2022; Marti [18]	N/A	N/A	N/A	N/A	N/A	N/A

**Table 4:** Malignancy characteristics of patients who underwent operations. ci-NPWT, closed incision–negative pressure wound therapy; FIGO, International Federation of Gynecology and Obstetrics; N/A, not available.

Cancer Characteristics						
*Year; Author*	*Cancer Site*		*Disease Stage (FIGO)*		*Neoadjuvant Therapy*	
	*ciNWPT*	*Conventional Gauze*	*ciNWPT*	*Conventional Gauze*	*ciNWPT*	*Conventional Gauze*
2016; Lynam [14]	Cervix 0 Uterine corpus 9 (41%) Ovarian 6 (27%)	Cervix 19 (9%) Uterine corpus 53 (25%) Ovarian 54 (26%)	I 5 (23%) II 1 (5%) III 5 (23%) IV 5 (23%)	I 49 (24%) II 9 (4%) III 5 34 (16%) IV 23 (11%)	1 (5%)	16 (8%)
2020; Chambers [15]	N/A	N/A	I 17 (27%) II 4 (6%) III 24 (38%) IV 12 (19%)	I 39 (20%) II 7 (4%) III 90 (47%) IV 37 (19%)	Neoadjuvant Chemotherapy 14 (22%)	Neoadjuvant Chemotherapy 44 (23%)
2021; Leitao [16]	Ovary/fallopian tube/peritoneal cancer 203 (80%) Uterine cancer 37 (15%) Cervical cancer 4 (2%) Other 5 (2%)	Ovary/fallopian tube/peritoneal cancer 207 (82%) Uterine cancer 32 (13%) Cervical cancer 2 (1%) Other 5 (2%)	N/A	N/A	Prior radiation therapy exposure 7 (3%) Prior chemotherapy exposure 85 (33%)	Prior radiation therapy exposure 8 (3%) Prior chemotherapy exposure 79 (31%)
2021; Yin [17]	N/A	N/A	N/A	N/A	N/A	N/A
2022; Marti [18]	Ovarian cancer 33 (57%) Endometrial cancer 20 (34%)	Ovarian cancer 61 (72%) Cervical cancer 1 (1%) Endometrial cancer 20 (24%) Vulvar cancer 1 (1%)	I 11 (19%) II 4 (7%) III 25 (43%) IV 3 (5%)	I 13 (15%) II 10 (12%) III 44 (52%) IV 9 (11%)	N/A	N/A

**Table 5:** Postoperative outcomes after laparotomy. ci-NPWT; closed incision–negative pressure wound therapy; SSI, surgical site injection; N/A, not available.

Postoperative Outcomes (ciNWPT vs. Conventional Gauze)					
*Year; Author*	*SSI*	*Dehiscence*	*Seroma*	*Hematoma*	*Length of Stay (Mean Days)*
2016; Lynam [14]	1 (5%) vs. 15 (7%)	3 (14%) vs. 25 (12%)	2 (9%) vs. 6 (3%)	1 (5%) vs. 3 (1%)	6.22 vs. 5.25
2020; Chambers [15]	6 (9%) vs. 70 (36%)	0 (0%) vs. 9 (5%)	3 (5%) vs. 13 (7%)	3 (5%) vs. 3 (2%)	N/A
2021; Leitao [16]	16 (6%) vs. 17 (7%)	30 (12%) vs. 25 (10%)	11 (4%) vs. 14 (6%)	2 (1%) vs. 1 (0.4%)	N/A
2021; Yin [17]	3 (21%) vs. 6 (23%)	2 (14%) vs. 0 (0%)	N/A	N/A	N/A
2022; Marti [18]	1 (2%) vs. 16 (19%)	1 (2%) vs. 16 (19%)	2 (3%) vs. 16 (19%)	1 (2%) vs. 8 (9%)	6.16 vs. 8.86
